# Postcolonoscopy Appendicitis: A Delayed Complication

**DOI:** 10.7759/cureus.7716

**Published:** 2020-04-17

**Authors:** Alsadiq Al Hillan, Mujtaba Mohamed, Diane Chien, Abbas Alshami, Faizan Arif

**Affiliations:** 1 Internal Medicine, Jersey Shore University Medical Center, Neptune, USA; 2 Internal Medicine, Hackensack Meridian Health, Jersey Shore University Medical Center, Neptune, USA; 3 Internal Medicine, Dorrington Medical Associates, Houston, USA; 4 Emergency Medicine, Jersey Shore University Medical Center, Neptune, USA

**Keywords:** colonoscopy complications, postcolonoscopy appendicitis

## Abstract

Colonoscopy is a procedure that enables a physician (usually a gastroenterologist) to directly image and examine the entire colon. It has both diagnostic and therapeutic benefits with a relatively low morbidity rate. Complications have been well described in the literature. Nevertheless, it is necessary for operators to be aware of the rare complications of colonoscopy. Acute appendicitis is an unusually rare occurrence following a colonoscopy, and it can be easily confused with other complications of the procedure. Prompt recognition of this complication can lead to early, effective treatment, and delayed diagnosis can lead to serious results. We present a case of a 33-year-old man who underwent a routine colonoscopy with no intraoperative complication who presented with appendicitis two weeks later as a rare delayed side effect; such a delayed presentation has not been described in the literature previously. This case highlights that appendicitis should be considered in the differential diagnosis of right-sided lower abdominal pain following a colonoscopy.

## Introduction

Colonoscopy is a commonly performed procedure with diagnostic and therapeutic intents. It is the gold standard test for the diagnosis of colonic diseases, and up to one-third of patients report at least one minor transient gastrointestinal (GI) symptom after colonoscopy such as abdominal pain, nausea, vomiting, and bowel spasm [1]. 

Noninfectious complications include colonic perforation, bleeding, splenic injury, postpolypectomy syndrome, and cardiopulmonary adverse events. Infectious complications include bacteremia, cholecystitis, and diverticulitis [2,3]. One extremely rare complication that has been less studied is postcolonoscopy appendicitis (PCA). We present the case of a 33-year-old man who underwent a routine colonoscopy with no intraoperative complication then presented with appendicitis two weeks later, highlighting a delayed side effect after the colonoscopy requiring intervention. 

## Case presentation

A 33-year-old man with a past medical history of ulcerative colitis underwent a routine colonoscopy where a benign polyp was found and removed with no complications. Approximately 15 days after the colonoscopy, the patient presented with lower mid-abdominal pain that radiated to the right lower quadrant. He described the pain as feeling like he needed to pass gas. Four days later, he arrived in the emergency room due to worsening pain. His vitals on admission showed a temperature of 98.8°F, a blood pressure of 129/66 mmHg, a heart rate of 80 beats/minute, and a respiratory rate of 18 cycles/minute. The white blood cell count was 11,100 cells/mm^3^; inflammatory markers including C-reactive protein and erythrocyte sedimentation rate were both unremarkable (Table 1)

**Table 1:** Labratory findings

	Result	Reference Range
White blood cell count	11.1 K/uL	4.5-11 K/uL
Hemoglobin	15.1 gm/dL	12-16 gm/dL
Hematocrit	44.1%	35%-48%
Platelet count	453 K/uL	140-450 K/uL
Blood urea nitrogen	11 mg/dL	5-25 mg/dL
Creatinine	0.8 mg/dL	0.44-1 mg/dL
Erythrocyte sedimentation rate	12 mm/hr	15-20 mm/hr
C-reactive protein	0.52 mg/L	< 1 mg/L

**Figure 1:**
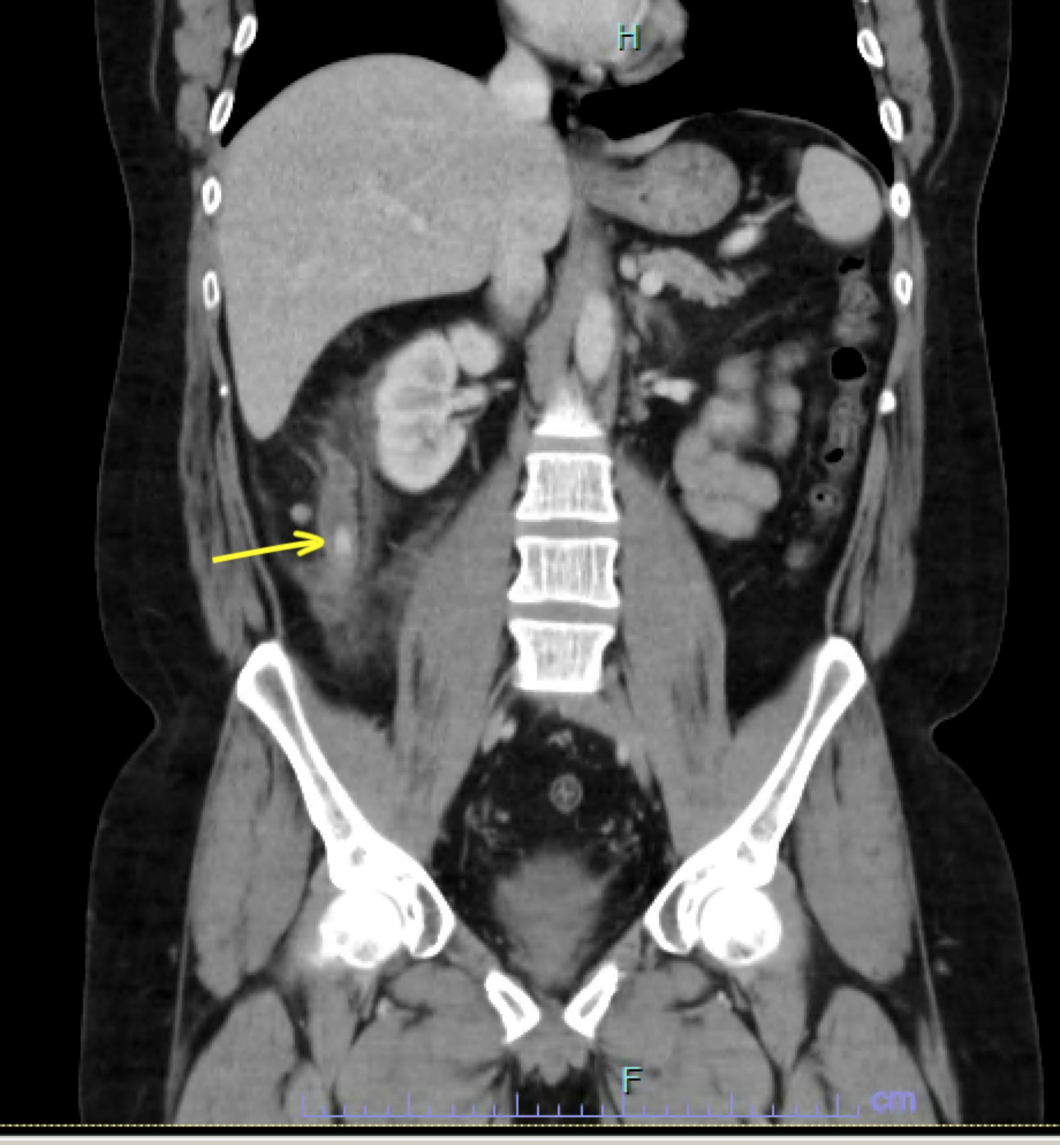
CT scan of the abdomen and pelvis showing acute appendicitis and an 11-mm appendicolith at the base of the appendix

Physical exam showed tenderness of the right lower quadrant without guarding. Axial computed tomography of the abdomen and pelvis with contrast showed dilated appendix measuring 21 mm with periappendiceal stranding consistent with acute appendicitis. The base of the appendix showed appendicolith, which is a calcified deposit within the appendix, 11 mm in diameter. There were no coexisting findings of colitis suggesting a flare-up (Figure 1)

There was no evidence of an abscess. The patient underwent a laparoscopic appendectomy, which was converted to an open appendectomy due to significant adherence of the appendix to the retroperitoneum. Surgery was successful after the open appendectomy, and the patient is currently recovering well. 

## Discussion

Colonoscopy is useful as a diagnostic and therapeutic tool for colorectal disease. It is a relatively safe procedure, but it has some risks, and major complications include bleeding and perforation. Minor complications may occur more commonly and include abdominal pain, vomiting, nausea, intestinal spasm, and mucosal tears in the lining of the colon [1]. Rare complications of a colonoscopy include mesenteric ischemia, cholecystitis, pancreatitis, appendicitis, small bowel perforation, volvulus, obstruction, and strangulation [2,3]. 

PCA is thought to be mainly caused by luminal obstruction, like the so-called barium appendicitis after a barium enema, and this can occur by obstruction of the appendiceal orifice via the retention of barium [3-5]. The suggested mechanisms of appendicitis after colonoscopy are pre-existing subclinical infection/inflammation of the appendix and elevated intraluminal pressure from colonoscopy air insufflation [6]. When intraluminal pressure is elevated, insufflated air can obstruct the luminal orifice by lengthening the appendix, and bowel contents and fecaliths (such as the case in our patient) can be forced into the appendix [7]. Additional mechanisms of appendicitis after colonoscopy are direct intubation of the colonoscope into the appendiceal lumen, which can induce inflammation, and mucosal injury around the appendiceal orifice, which can cause local edema and obstruction of the appendiceal lumen [6-8].

Acute appendicitis after lower GI endoscopy has an estimated incidence of 3.8 to 4.9 per 10,000 colonoscopies [6,9]. The incidence rate of acute appendicitis is significantly higher in the first week after colonoscopy [10]. The median age at presentations is 55 years (range, 24 to 84 years), with more male individuals being affected (n=37, 64.9%) [11].

The duration of symptoms can last zero to nine days [11]. Our patient presented two weeks after the operation as a delayed side effect, which is a very rare delayed onset that has never been described previously in the literature. Fecalith impaction into the appendiceal lumen, direct trauma, a pre-existing subclinical infection of the appendix, and underlying ulcerative colitis (as seen in our patient) seem to play an important role in the pathogenesis. Timely diagnosis and intervention are crucial to attaining a satisfactory outcome.

## Conclusions

Although PCA is a rare entity, it poses a diagnostic challenge given the similarity of presentation with other more well‐known complications. Physicians should always be aware of this rare complication, as an early recognition can lead to early, effective treatment, and delayed diagnosis can lead to serious outcomes.
